# A comparison of resource utilization following chemotherapy for acute myeloid leukemia in children discharged versus children that remain hospitalized during neutropenia

**DOI:** 10.1002/cam4.481

**Published:** 2015-06-24

**Authors:** Kelly D Getz, Tamara P Miller, Alix E Seif, Yimei Li, Yuan-Shung Huang, Rochelle Bagatell, Brian T Fisher, Richard Aplenc

**Affiliations:** 1 Division of Oncology, The Children's Hospital of PhiladelphiaPhiladelphia, Pennsylvania; 2 Center for Pediatric Clinical Effectiveness, The Children's Hospital of PhiladelphiaPhiladelphia, Pennsylvania; 3 Department of Pediatrics, University of Pennsylvania School of MedicinePhiladelphia, Pennsylvania; 4 Center for Clinical Epidemiology and Biostatistics, University of Pennsylvania School of MedicinePhiladelphia, Pennsylvania; 5 Division of Infectious Diseases, The Children's Hospital of PhiladelphiaPhiladelphia, Pennsylvania

**Keywords:** Acute myeloid leukemia, neutropenia, patient discharge, pediatrics

## Abstract

Comparisons of early discharge and outpatient postchemotherapy supportive care in pediatric acute myeloid leukemia (AML) patients are limited. We used data from the Pediatric Health Information System on a cohort of children treated for newly diagnosed AML to compare course-specific mortality and resource utilization in patients who were discharged after chemotherapy to outpatient management during neutropenia relative to patients who remained hospitalized. Patients were categorized at each course as early or standard discharge. Discharges within 3 days after chemotherapy completion were considered “early”. Resource utilization was determined based on daily billing data and reported as days of use per 1000 hospital days. Inpatient mortality, occurrence of intensive care unit (ICU)-level care, and duration of hospitalization were compared using logistic, log-binomial and linear regression methods, respectively. Poisson regression with inpatient days as offset was used to compare resource use by discharge status. The study population included 996 patients contributing 2358 treatment courses. Fewer patients were discharged early following Induction I (7%) than subsequent courses (22–24%). Across courses, patients discharged early experienced high readmission rates (69–84%), yet 9–12 fewer inpatient days (all *P* < 0.001). Inpatient mortality was low across courses and did not differ significantly by discharge status. The overall risk for ICU-level care was 116% higher for early compared to standard discharge patients (adjusted risk ratio: 2.16, 95% confidence interval: 1.50, 3.11). Rates of antibiotic, vasopressor, and supplemental oxygen use were consistently elevated for early discharge patients. Despite similar inpatient mortality to standard discharge patients, early discharge patients may be at greater risk for life-threatening chemotherapy-related complications, including infections.

## Introduction

Treatment for pediatric acute myeloid leukemia (AML) involves multiple consecutive courses of intensive chemotherapy followed by periods of prolonged severe neutropenia with substantive infection risks. Current supportive care guidelines in the Children's Oncology Group (COG) Phase III AML trials recommend hospitalization for the duration of chemotherapy and associated marrow aplasia. However, a recent survey of pediatric supportive care practices suggests that up to one-third of pediatric AML patients are discharged early to outpatient management during postchemotherapy neutropenia 1. While outpatient supportive care in this population has only been examined in a single study of 26 patients 2, several studies of adult AML patient populations suggest that outpatient management during neutropenia may be safe and feasible 1–11. Early hospital discharge was associated with fewer febrile episodes 3,11, reduced use of intravenous antibiotics 3,5,8,9 and comparable mortality 2,11. The studies to date generally reported on a single institution, were limited by small sample sizes, did not adjust for potential confounding, or lacked an appropriate inpatient reference population. To address these limitations, we used data from a large nationally representative cohort of children with new onset AML assembled using the Pediatric Health Information System (PHIS) database 12. Course-specific mortality and resource utilization in pediatric patients who were discharged postchemotherapy to outpatient management during neutropenia were compared to similar patients who remained hospitalized. Based on the aforementioned data in adult AML populations, we hypothesized that early discharge would be associated with fewer in-hospital days per chemotherapy course with no increase in inpatient mortality or resource utilization.

## Methods

### Data source

PHIS is an administrative database that contains inpatient, emergency department, ambulatory surgery, and observation unit information from 46 not-for-profit, tertiary care pediatric hospitals representing 17 large metropolitan areas, and accounts for nearly half of all pediatric discharges in the United States 13. Data include demographics, dates of service, discharge disposition, and daily inpatient billing data for medications, laboratory tests, imaging procedures, clinical services, and supplies. Patients are assigned a unique identifier which allows records to be linked longitudinally across admissions. Data are anonymized at the time of submission and subject to a number of reliability and validity checks before inclusion in the database. Data quality is assured through a joint effort between the Children's Hospital Association and participating hospitals.

### Study population

The current study population was derived from a cohort of children treated for new onset AML previously assembled from PHIS data 12. Briefly, patients with a discharge diagnosis for any myeloid or unspecified leukemia (International Classification of Diseases-9 codes 205.xx–208.xx) following an admission occurring from January 1999 through March 2010 were identified in the PHIS database. Those with a diagnosis for an alternative malignancy or an indication of bone marrow transplantation within 60 days of the first diagnosis admission were excluded. Daily pharmacy data for each patient were then manually reviewed and chemotherapy administration patterns were matched to conventional pediatric AML treatment regimens. Patients who did not receive induction chemotherapy consistent with AML therapy were excluded.

The match quality for each regimen was rated as perfect, excellent (i.e., deviation of only 1 day for any of the medications in the regimen), probable/identifiable (i.e., deviation of more than 1 day for any of the medications in the regimen), or unknown (i.e., regimen did not match any conventional AML regimen). For these analyses, course-specific cohorts were limited to patients who received standard chemotherapy defined as a perfect or excellent chemotherapy match to the following regimens: ADE (cytarabine, daunorubicin, etoposide, ±gemtuzumab) at induction courses, AE (cytarabine, etoposide) at Intensification I, and MA (mitoxantrone, cytarabine, ±gemtuzumab) at Intensification II. If a patient received chemotherapy that was inconsistent with the regimens defined above, the patient was excluded from the analyses for that course and any subsequent courses.

The population of patients at each treatment course was further restricted to those considered eligible for discharge postchemotherapy and prior to absolute neutrophil count (ANC) recovery based upon a review of daily hospital resource utilization data. Within a course, patients were considered discharge-eligible if there was no record of ICU-level care or blood culture from the first day of chemotherapy through the last day of chemotherapy plus 3 days and there was no record of vasopressor use or supplemental oxygen therapy within the 3 days after the last day of chemotherapy. As previously detailed 14, ICU-level care was defined by the occurrence-specific ICD-9-CM procedure codes or clinical resources considered a priori as a marker of ICU care, rather than by physical location.

### Exposure

For each patient, dates of initial discharge following the last day of chemotherapy administration were determined at each course. The timing of discharge relative to the last day of chemotherapy in a given course was calculated as the difference between the dates of discharge and the last chemotherapy administration. Discharges occurring within the 3 days after chemotherapy completion were categorized as “early discharge” while those discharged more than 3 days postchemotherapy completion and those not discharged before the start of the next treatment course were categorized as “standard discharge.”

### Outcomes

Course-specific follow-up started 4 days after the last day of chemotherapy and continued until the earliest of the following: the start of the subsequent course defined as the first inpatient day on which chemotherapy was administered, death, or 50 days after commencement of chemotherapy. If patients were discharged and subsequently readmitted within the follow-up period, then data for these readmissions were included in the analyses.

Outcomes of interest included the total number of inpatient days, the number of days to the start of the next treatment course, inpatient case fatality, and receipt of any ICU-level care. Additionally, utilization rates of the following resources were documented during the follow-up period: antibiotic, antifungal, antiviral and vasopressor medications, parenteral nutrition, blood product replacement, and supplemental oxygen. Inpatient case fatality rates were calculated based on discharge status which is coded for each hospitalization. Daily billing data were used to identify patients receiving any ICU-level care and to assess rates of utilization for the individual resources specified above. Binary indicator variables were created for each of the resources to designate exposure on each inpatient day and were summed to obtain the total number of days exposed. Resource utilization rates were reported as days of use per 1000 inpatient days. Each of the outcome measures was compared within chemotherapy courses by discharge status (i.e., early discharge versus standard discharge). For the subpopulation of patients who were discharged early, rates and timing of first readmission relative to the start of follow-up were also computed.

### Covariates

Patient characteristics including age (categorized as <1, 1 to <5, 5 to <10, 10 to <15, and ≥15 years), sex, race (dichotomized as white, non-white), and insurance status at the start of each chemotherapy course (categorized as private, government, other) were ascertained from PHIS. A hospital-level variable representing a hospital's early discharge practice was created based on the overall proportion of patients discharged early across courses.

### Statistical analyses

Histograms of the timing of discharge relative to the completion of chemotherapy were plotted for each course. The frequencies of early discharge (*n*, %) were tabulated by course and patient characteristics and were compared using chi-squared tests. A plot of hospital-specific rates of early discharge was generated to assess variability in practice. Linear regression models were used to compare continuous outcomes, specifically the total number of inpatient days and days to next chemotherapy course, by discharge status. Course-specific case fatality rates (*n*, %) were compared for early versus standard discharge status using logistic regression methods which provide a reliable approximation of the risk ratio for binary outcome variables when the event of interest is sufficiently rare (i.e., incidence proportion <10%) 15. The odds ratio is a less reliable estimate of the corresponding risk ratio when the event of interest is more common. Thus, log-binomial regression methods were used to directly estimate the adjusted risk ratios (aRR) and corresponding 95% confidence intervals (CI) comparing occurrence of any ICU-level care by discharge status 16. Poisson regression models with inpatient days as offset were used to estimate adjusted rate ratios (aIRR) and 95% CI comparing counts of the number of days of use for each resource by discharge status. The log of total number of inpatient days was included as an offset to account for the different observation periods for different subjects and a Pearson scale adjustment was employed to correct for possible overdispersion. All multivariate models were adjusted for the covariates defined above.

## Results

### Study population

**Figure 1:**
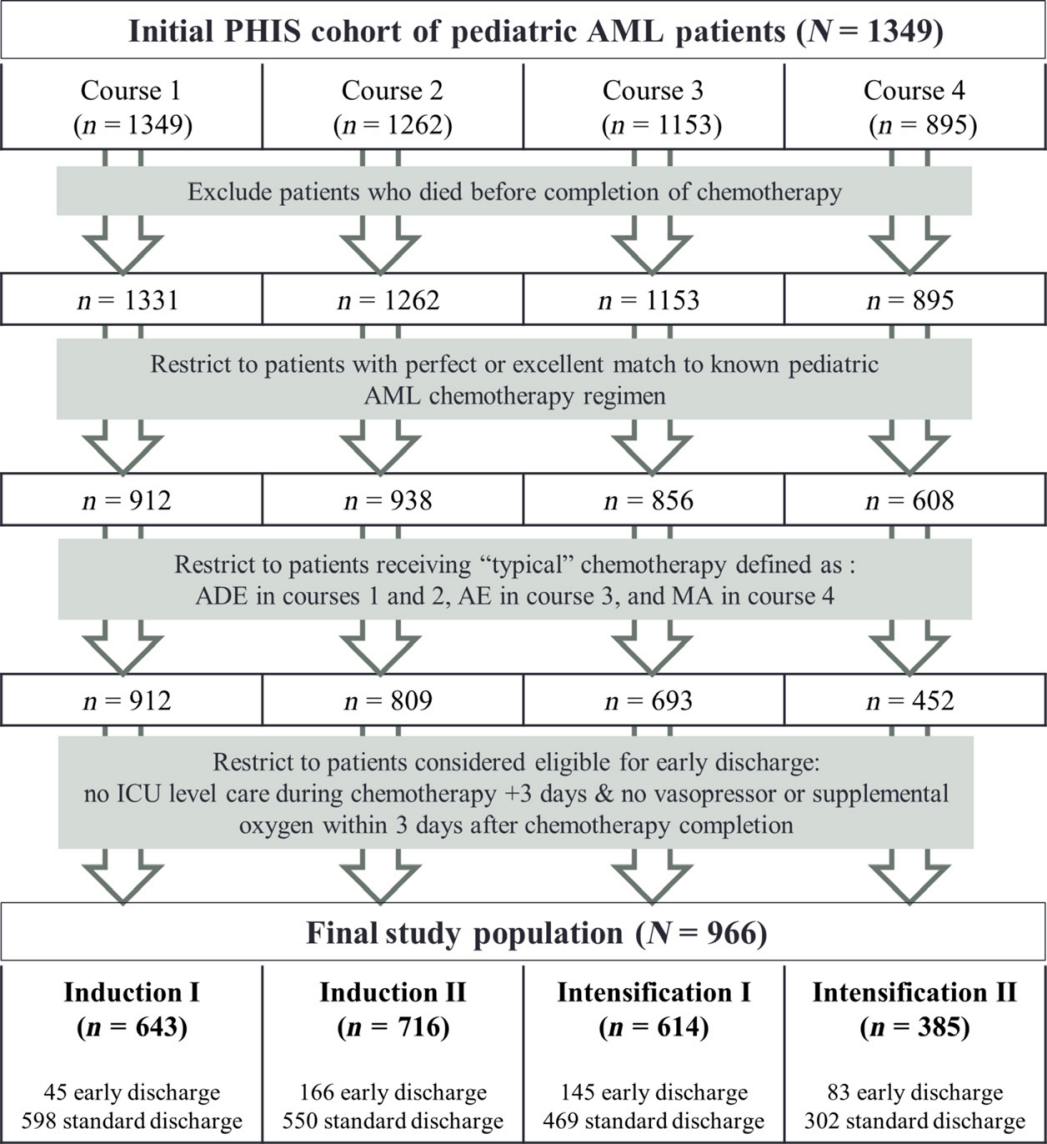
Flowchart depicting assembly of study population from established PHIS cohort of newly diagnosed pediatric acute myeloid leukemia patients. PHIS, Pediatric Health Information System database; AML, acute myeloid leukemia; ADE, cytarabine, daunorubicin, etoposide, ±gemtuzumab; AE, cytarabine and etoposide; MA, mitoxantrone, cytarabine, ±gemtuzumab.

A flow chart depicting the assembly of the study population is presented in Figure1. The initial PHIS cohort included 1349 patients with new onset AML who were treated with ADE at Induction I. Of these patients approximately 72% (*n* = 966) met all inclusion criteria to be included in the current analyses for at least one treatment course and contributed a total of 2358 chemotherapy courses. Patient characteristics for this population are presented in Table1. Overall, there were no significant differences in baseline characteristics between early and standard discharge patients.

**Table 1:** Baseline demographics of AML patients

		Discharge status		
	Overall (*N *= 966)	Early^1^(*n *= 243)	Standard^2^(*n *= 753)	*P*-value
Age, *n* (%)				0.8002
<1 years	118 (12.2)	29 (11.9)	89 (11.8)	
1 to <5 years	245 (25.4)	87 (35.8)	187 (24.8)	
5 to <10 years	162 (16.8)	132 (54.3)	117 (15.5)	
10 to <15 years	272 (28.2)	199 (81.9)	205 (27.2)	
≥15 years	199 (20.6)	243 (100)	155 (20.6)	
Gender, *n* (%)				0.2637
Male	510 (52.8)	132 (54.3)	378 (50.2)	
Female	486 (50.3)	111 (45.7)	375 (49.8)	
Race, *n* (%)				0.4671
White	653 (67.6)	164 (67.5)	489 (64.9)	
Non-white	343 (35.5)	79 (32.5)	264 (35.1)	
Insurance, *n* (%)				0.1348
Private	380 (39.3)	105 (43.2)	275 (36.5)	
Public	426 (44.1)	99 (40.7)	327 (43.4)	
Other	190 (19.7)	39 (16)	151 (20.1)	

1 Includes patients who were discharged early following one or more treatment courses.

2 Includes patients who were never discharged early.

### Distribution of timing of discharge after chemotherapy

**Figure 2:**
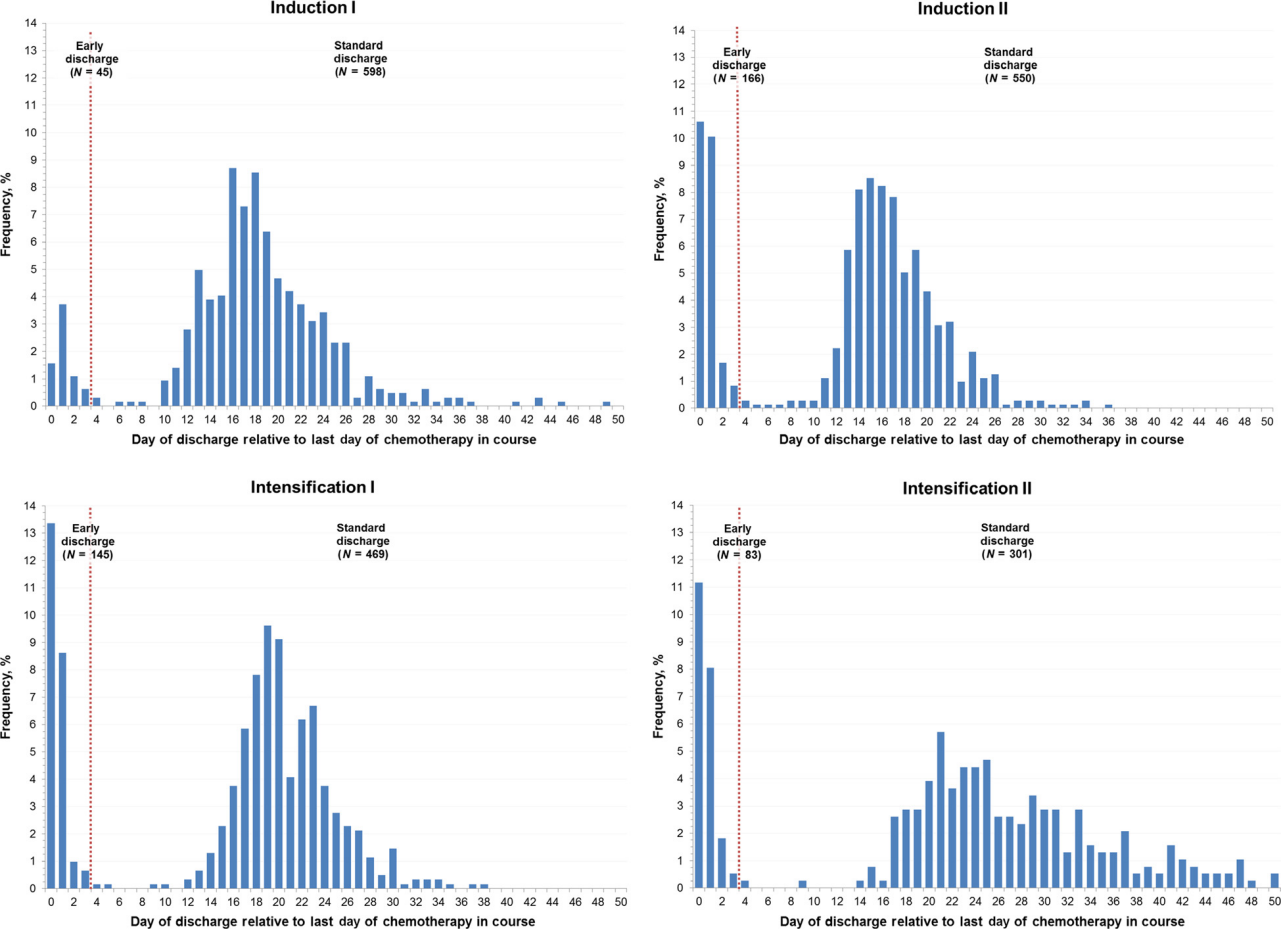
Distribution of timing of discharge relative to the last day of chemotherapy for each course. The vertical red stippled line transects each plot at the study defined threshold for early discharge.

Figure2 illustrates the bimodal distribution of timing of discharge relative to the completion of chemotherapy at each course. A subset of patients were discharged within a few days of their last chemotherapy administration at each course, but most patients remained hospitalized with median times to discharge ranging from 15 to 23 days postchemotherapy completion across treatment courses (Table2). A small number of patients following each chemotherapy course remained hospitalized through the start of the next course; this was more common following Induction I (13.5%) than following later courses (2.3–4.5%).

**Table 2:** Days to discharge, frequency of early discharge, and rate and timing of readmission after early discharge by treatment course

	Induction I (*N*=643)	Induction II (*N*=716)	Intensification I (*N*=614)	Intensification II (*N*=385)
Days to discharge, median (range)	18 (0–49)	15 (0–36)	19 (0–38)	23 (0–50)
Early discharge, *n* (%)	45 (7.0)	166 (23.2)	145 (23.6)	83 (21.6)
Readmission, *n* (%)	38 (84.4)	124 (74.7)	100 (69.0)	58 (69.9)
Days to first readmission, median (range)	4.0 (1.0–15.0)	7.0 (1.0–24.0)	8.0 (1.0–29.0)	7.0 (2.0–13.0)

### Rates of early discharge

The frequencies of early discharge along with rates and timing of readmission are presented in Table2 by course. Rates of early discharge varied by course, with early discharge being significantly less likely following Induction I (7.0%) than following subsequent courses (21.6–23.6%; all *P* < 0.0001). Readmission rates following early discharge were high and ranged from 69% to 84% across courses. Median time to first readmission was somewhat shorter for Induction I (4 days) compared to other courses (7–8 days) (all *P* < 0.001).

**Figure 3:**
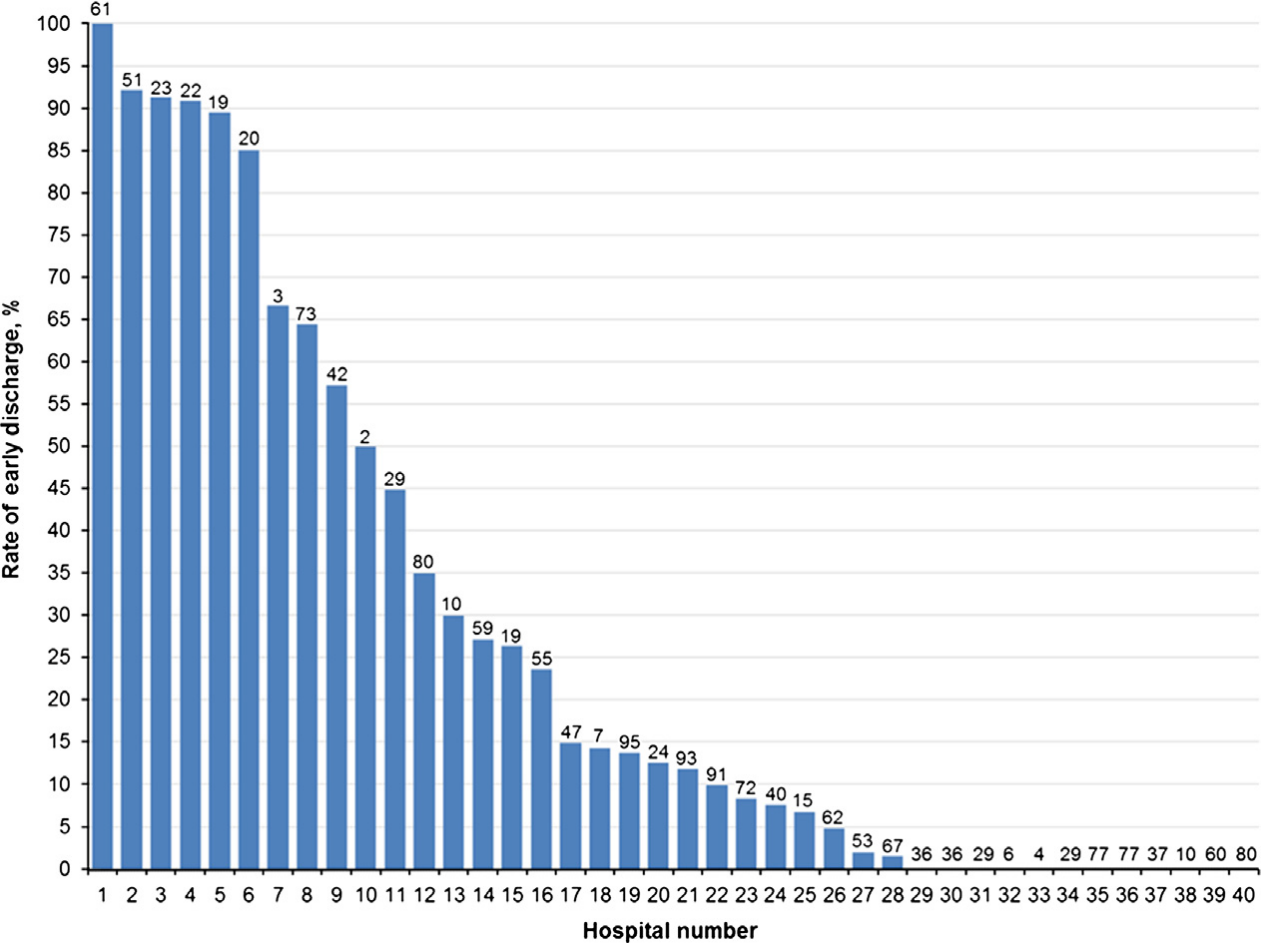
Variation in rates of early discharge by hospital. Hospitals are numbered in order of decreasing rate of early discharge. The numbers above each bar indicate the number of courses contributed by the hospital.

Rates of early discharge by patient characteristics are presented in Table S1. Across courses, rates of early discharge did not differ significantly by age, sex or race. However, patients with public or private insurance had higher rates of early discharge than those with other types of coverage at Intensification I (29.6% and 23.1% versus 11.5%, *P* = 0.0008) and Intensification II (24.0% and 24.9% versus 5.9%, *P* = 0.0043). As illustrated in Figure3, hospital-specific rates of early discharge varied substantially ranging from 0% to 100%. There was little intrapatient variability in the timing of discharge between courses excluding Induction I. Among patients who were discharged early after Induction II, 83% were also discharged early following subsequent courses. Among those who remained in the hospital after Induction II, 93% were consistently managed as an inpatient for subsequent courses.

### Comparison of outcomes by early versus standard discharge status

Comparisons of total inpatient days, inpatient mortality, ICU-level care, and time to the next course by discharge status are also presented in Table3. Despite high rates of readmission, patients discharged early experienced approximately 9–12 fewer inpatient days across treatment courses (all *P* < 0.001). Mortality rates following each course were generally low (0–2%) and did not differ significantly by early versus standard discharge status. However, more early discharge patients required any ICU-level care (7.2–18.1%) compared to standard discharge patients (2.0–8.6%) across all chemotherapy courses with the exception of Induction I (aRR: 2.16, 95% CI: 1.50, 3.11). The proportion of patients requiring ICU-level care increased with successive courses from Induction II to Intensification II for both early (*P* = 0.011) and standard discharge (*P* < 0.0001). Additionally, mean times to next treatment course were longer for intensification than induction courses (*P* < 0.0001) but did not differ for early versus standard discharge patients.

**Table 3:** Comparisons of total inpatient days, inpatient mortality, ICU-level care, and time to next course by discharge status

	Discharge status		Adjusted comparison^1^^,^^2^(95% CI)	*P*-value	Discharge status		Adjusted comparison^1^^,^^2^(95% CI)	*P*-value
	Early	Standard			Early	Standard		
	Induction I				Induction II			
Total inpatient days, median (range)	11.0 (1–39)	20.0 (6–42)	−9.2 (−11.2, −7.2)	<0.0001	9.5 (1–42)	18.0 (5–43)	−8.58 (−9.6, −7.6)	<0.0001
Inpatient mortality, *n* (%)	0 (0)	2 (0.30)	NE		2 (1.2)	3 (0.6)	2.79 (0.42, 18.6)	0.291
ICU-level care, *n* (%)	2 (4.4)	22 (3.7)	1.64 (0.38, 7.12)	0.507	12 (7.2)	11 (2.0)	4.55 (2.00, 10.4)	0.0003
Days to next course, mean (SD)	26.6 (9.0)	29.0 (9.1)	−2.44 (−5.25, 0.36)	0.089	28.6 (9.8)	29.5 (9.4)	−0.91 (−2.62, 0.80)	0.298
	Intensification I				Intensification II			
Total inpatient days, median (range)	10.0 (1–42)	22.0 (11–47)	−11.6 (−12.8, −10.5)	<0.0001	20.0 (1–56)	28.0 (13–61)	−8.8 (−10.9, −6.7)	<0.0001
Inpatient mortality, *n* (%)	0	0	NE		2 (2.4)	2 (0.7)	2.81 (0.36, 21.8)	0.323
ICU-level care, *n* (%)	15 (10.3)	24 (5.1)	2.29 (1.19, 4.42)	0.0132	15 (18.1)	26 (8.6)	2.42 (1.26, 4.66)	0.0081
Days to next course, mean (SD)	38.4 (38.3)	38.1 (29.5)	1.04 (−5.60, 7.67)	0.759	49.6 (30.6)	52.9 (33.2)	−2.28 (−11.2, 6.61)	0.614

CI, confidence interval; ICU, intensive care unit; SD, standard deviation; NE, not estimable.

1 Mean difference presented for total inpatient days and days to next course. Odds ratio presented for inpatient mortality. Risk ratio presented for ICU-level care.

2 Due to the small numbers of deaths crude associations are presented for inpatient mortality. All other comparisons are adjusted for patient age at diagnosis, race, sex, and insurance status.

Table4 presents comparisons of resource utilization rates by discharge status for each treatment course. Rates of inpatient antibiotic utilization were consistently higher among patients discharged early than among those who remained hospitalized, with an average increase of 43% across courses (aIRR: 1.43, 95%:1.36, 1.51). Rates of vasopressor (aIRR: 4.85, 95% CI: 3.05, 7.71) and supplemental oxygen (aIRR: 2.88, 95% CI: 1.83, 4.52) utilization were also higher among early discharge patients compared to standard discharge patients across all treatment courses. Overall blood product replacement rates were elevated among those discharged early only for Induction I (aIRR: 1.29, 95% CI: 1.09, 1.54) and Intensification II (aIRR: 1.25, 95% CI: 1.12, 1.40) chemotherapy courses. Although utilization rates of antifungal agents overall were comparable for early and standard discharge patients, there was a trend toward greater use of amphotericin products in the early discharge group which was offset by greater use of other antifungal agents in the standard discharge group (Table S2). There were no significant differences in the rates of total parenteral nutrition and antiviral agent utilization between the early and standard discharge patients at any course.

**Table 4:** Comparisons of resource utilization rates (per 1000 inpatient days) by discharge status for each treatment course

	Discharge status			Discharge status		
	Early	Standard	aIRR (95% CI)	Early	Standard	aIRR (95% CI)
	Induction I			Induction II		
Antibiotics	1698.0	1404.4	1.30 (1.13, 1.50)*	1620.5	996.5	1.60 (1.46, 1.76)*
Antifungals	848.5	889.5	0.91 (0.74, 1.12)	814.8	857.4	0.97 (0.89, 1.07)
Antivirals	70.7	145.6	0.59 (0.21, 1.62)	114.8	151.2	0.67 (0.39, 1.13)
Vasopressors	27.6	5.2	8.07 (3.50, 18.6)*	32.7	6.8	4.54 (2.08, 9.89)*
Blood Products	298.8	246.5	1.29 (1.09, 1.54)*	238.7	221.5	1.06 (0.94, 1.19)
Platelets	206.1	179.5	1.32 (1.05, 1.66)*	150.2	144.5	1.03 (0.88, 1.21)
Packed RBC	135.2	105.7	1.30 (1.06, 1.59)*	144.9	106.8	1.20 (1.06, 1.36)*
FFP	6.8	4.9	1.41 (0.44, 4.56)	12.4	3.3	3.03 (1.23, 7.48)*
Parenteral Nutrition	111.4	169.8	1.00 (0.50, 2.01)	86.2	84.8	1.28 (0.76, 2.14)
Oxygen therapy	40.9	13.3	5.14 (1.96, 13.5)*	32.2	8.7	3.63 (1.79, 7.36)*
	Intensification I			Intensification II		
Antibiotics	1589.9	1030.4	1.52 (1.39, 1.67)*	1862.1	1357.5	1.36 (1.25, 1.49)*
Antifungals	775.3	853.1	0.93 (0.84, 1.03)	882.9	915.9	0.96 (0.87, 1.07)
Antivirals	116.2	170.1	0.70 (0.41, 1.19)	109.0	165.4	0.62 (0.32, 1.21)
Vasopressors	40.6	9.2	5.67 (2.63, 12.2)*	45.7	14.0	3.56 (1.65, 7.66)*
Blood products	259.3	228.7	1.17 (0.99, 1.38)	383.1	319.6	1.25 (1.12, 1.40)*
Platelets	168.3	154.5	1.21 (0.96, 1.52)	287.0	244.2	1.26 (1.09, 1.46)*
Packed RBC	151.4	109.6	1.42 (1.18, 1.72)*	171.5	133.8	1.32 (1.11, 1.56)*
FFP	5.6	2.2	4.31 (1.61, 11.5)*	8.6	5.5	1.72 (0.56, 5.25)*
Parenteral Nutrition	73.7	99.4	1.22 (0.75, 1.98)	109.0	88.3	1.23 (0.82, 1.85)
Oxygen Therapy	40.0	18.3	2.22 (1.10, 4.51)*	46.3	16.2	2.85 (1.39, 5.83)*

All comparisons adjusted for patient age at diagnosis, race, sex, and insurance status at start of course. aIRR, adjusted rate ratio; CI, confidence interval; RBC, red blood cells; FFP, fresh frozen plasma.

* *P* < 0.05.

## Discussion

Our study found that pediatric AML patients who were discharged early following induction and intensification chemotherapy courses had similar course-specific survival, fewer days of hospitalization, and no apparent delay in progression to subsequent treatment courses compared to standard discharge patients. However, early discharge patients were frequently readmitted and had higher rates of antibiotic, vasopressor, and supplemental oxygen utilization than patients who remained hospitalized. Together these findings suggest that early discharge patients may have an increased risk for life-threatening infectious complications compared to standard discharge patients, but they are successfully treated upon readmission.

We observed significant variability in practice across hospitals with respect to postchemotherapy neutropenia management strategies. For some hospitals the majority of patients were identified as early discharge patients, while others maintained hospitalization for most, if not all, AML patients. This variation in practice is consistent with a recent survey of supportive care approaches among COG institutions 1 and likely reflects the dearth of studies to compare the effectiveness of the two strategies in pediatric AML populations. One pediatric study found similar rates of mortality for outpatient versus inpatient management of neutropenia following AML induction chemotherapy 2 which is consistent with our findings. Another study evaluating outpatient versus inpatient AML intensification chemotherapy found fewer days of febrile neutropenia and less antimicrobial use among outpatients than children receiving inpatient treatment 17. Likewise, previous evaluations among adult AML patients found outpatient supportive care to be associated with fewer and shorter febrile episodes 5,9 and fewer days of intravenous antibiotic administration 3,5,9. The findings with respect to antimicrobial agent use contradict those of the current study. However, these previous studies have had a number of limitations which may have resulted in biased associations: most included data from only a single institution, had small sample sizes, lacked an internal reference or used an inpatient reference population which included patients too ill to be discharged, or did not adjust resource use comparisons for differences in duration of hospitalization between compared groups.

Our study has several strengths over the previous pediatric and adult publications. Our analysis was performed using a large nationally representative cohort of pediatric AML patients, increasing the generalizability of our results over small single institution studies. Also, unlike most previous evaluations, we restricted our comparison of early versus standard discharge to include only AML patients that met eligibility criteria for early discharge at the time of completion of chemotherapy. In the absence of this restriction, the standard discharge reference population would likely include patients with poorer baseline risk than those actually discharged to outpatient supportive care which would lead to biased comparisons.

Despite the strengths of the study, our results should be considered in light of some potential limitations. Billing data on resource utilization rather than medical record data were used to identify patients as discharge-eligible. Thus, it is possible that some higher risk patients who were not actually eligible for early discharge were retained in the analyzed cohort. However, more of these patients would likely be included in the standard than the early discharge group, which would bias observed associations toward no effect. The classification of early discharge was not informed by ANC measurements, but the threshold for early discharge was 3 days after completing chemotherapy; neutrophil recovery within this timeframe is improbable. Therefore, it is expected that all early discharge patients were neutropenic at the time of discharge. We also restricted the study population to patients whose chemotherapy courses were reliably matched to conventional AML regimens, which would further minimize bias in the classification of relative timing of discharge. We may also be missing information on resource utilization if patients were readmitted after being discharged to a hospital other than the one in which chemotherapy was delivered or if outpatient rather than inpatient resources were utilized. This pattern of missing data is likely differential leading to a greater underestimate of resource utilization in the early discharge patients than the standard discharge patients, suggesting that the increases observed following early discharge for some of the resources may be conservative estimates. Since our data were limited to inpatient resource utilization, we were unable to ascertain whether patients discharged early were prescribed antibacterial prophylaxis. Previous data suggest that a limited number of institutions utilize prophylactic antibiotics in patients with AML 1, thus we expect the proportion of patients to be similarly low (<13%) in the current study population. Others have reported reduced infection rates with intravenous versus oral or no prophylactic antibiotic administration among pediatric AML patients discharged after chemotherapy to outpatient management during neutropenia 18. Thus, it is possible that the detrimental effects that we observed in relation to early discharge may be attenuated among those patients utilizing such antibacterial prophylaxis. Increased rates of ICU-level care, antibiotic, vasopressor and supplemental oxygen utilization were assumed to be a marker for an increased incidence of clinically relevant infection in the early discharge patients. The absence of clinical and laboratory data for infection did not allow for confirmation of this assumption.

In summary, these data suggest that outpatient supportive care achieves fewer inpatient days per course and similar course-specific survival relative to inpatient supportive care. However, patients discharged early to outpatient management during neutropenia may be at greater risk for infectious complications that upon readmission require more intensive care resources for effective management. Additional work is required to ascertain the specific microbiological organisms involved, to compare the costs of early versus standard discharge strategies, and to assess patient and caregiver preferences about the respective advantages and disadvantages of the two strategies of postchemotherapy neutropenia management.

## Conflict of Interest

B. T. F. reported research funding from Merck and Pfizer unrelated to this project. The remaining authors declare no competing financial interests.

## Supporting Information

Table S1. Rates of early discharge by patient characteristics. **Table S2.** Comparisons of resource utilization rates (per 1000 inpatient days) by discharge status for each treatment course.
