# Supplementary material for: A comparison of resource utilization following chemotherapy for acute myeloid leukemia in children discharged versus children that remain hospitalized during neutropenia
Source: Cancer Med. 2015 Jun 24;4(9):1356–64. doi: 10.1002/cam4.481 (PMC4567020; doi:10.1002/cam4.481)
Supplement: Table S1. — Rates of early discharge by patient characteristics. Table S2. Comparisons of resource utilization rates (per 1000 inpatient days) by discharge status for each treatment course. [file cam40004-1356-sd1.docx]

**Supporting Information**

**eTable 1. Rates of early discharge by patient characteristics**

|  |  | **Induction I** | | **Induction II** | | **Intensification I** | | **Intensification II** | |
| --- | --- | --- | --- | --- | --- | --- | --- | --- | --- |
| **Patient Characteristic** | | **Early Discharge** | | **Early Discharge** | | **Early Discharge** | | **Early Discharge** | |
|  |  | **n (%)** | **p-value^a^** | **n (%)** | **p-value^a^** | **n (%)** | **p-value^a^** | **n (%)** | **p-value^a^** |
| **Age, years** | |  |  |  |  |  |  |  |  |
|  | <1 | 4 (5.9) | 0.3896 | 16 (19.2) | 0.8209 | 19 (25.0) | 0.9016 | 12 (25.5) | 0.7003 |
|  | 1 - <5 | 10 (6.1) |  | 41 (22.5) |  | 38 (23.8) |  | 24 (22.4) |  |
|  | 5- <10 | 13 (11.3) |  | 31 (26.3) |  | 27 (26.7) |  | 10 (17.0) |  |
|  | 10 - <15 | 11 (6.6) |  | 48 (24.1) |  | 37 (21.6) |  | 26 (23.9) |  |
|  | ≥15 | 7 (5.4) |  | 30 (22.4) |  | 24 (22.6) |  | 11 (17.5) |  |
| **Gender** | |  |  |  |  |  |  |  |  |
|  | Male | 21 (6.5) | 0.6442 | 94 (25.6) | 0.1143 | 76 (24.9) | 0.4503 | 45 (23.7) | 0.3167 |
|  | Female | 24 (7.6) |  | 72 (20.6) |  | 69 (22.3) |  | 38 (19.5) |  |
| **Race** | |  |  |  |  |  |  |  |  |
|  | White | 34 (7.9) | 0.2589 | 114 (23.9) | 0.7467 | 107 (25.6) | 0.0694 | 56 (22.0) | 0.4429 |
|  | Non-white | 11 (5.8) |  | 48 (22.1) |  | 34 (18.3) |  | 23 (18.6) |  |
| **Insurance** | |  |  |  |  |  |  |  |  |
|  | Private | 25 (9.8) | 0.0754 | 62 (22.1) | 0.8387 | 74 (29.6) | 0.0008 | 37 (24.0) | 0.0043 |
|  | Public | 14 (5.0) |  | 70 (24.1) |  | 58 (23.1) |  | 43 (24.9) |  |
|  | Other^b^ | 6 (5.4) |  | 34 (23.5) |  | 13 (11.5) |  | 3 (5.9) |  |
| ^a^ p-value comparing early discharge rates across levels of covariates  ^b^ Other category includes self-pay, other, and unknown | | | | | | | | | |

**eTable 2. Comparisons of resource utilization rates (per 1000 inpatient days) by discharge status for each treatment course**

|  |  | **Induction I** | | | **Induction II** | | |
| --- | --- | --- | --- | --- | --- | --- | --- |
|  |  | Discharge Status | |  | Discharge Status | |  |
|  |  | Early | Standard | aIRR (95% CI) | Early | Standard | aIRR (95% CI) |
| Antibiotics, total of 5 subclasses | | 1698.0 | 1404.4 | 1.30 (1.13, 1.50)* | 1620.5 | 996.5 | 1.60 (1.46, 1.76)* |
|  | Beta lactam anti-*Pseudomonas* | 706.0 | 657.8 | 1.58 (1.22, 2.06)* | 751.9 | 459.0 | 2.24 (1.93, 2.60)* |
|  | Broad Gram-positive | 478.9 | 372.8 | 1.98 (1.50, 2.62)* | 487.9 | 289.7 | 2.27 (1.90, 2.71)* |
|  | Aminoglycosides | 277.7 | 179.7 | 1.78 (1.12, 2.82)* | 237.7 | 138.3 | 2.35 (1.76, 3.14)* |
|  | Carbapenems with anti-*Pseudomonas* activity | 204.2 | 171.9 | 2.29 (1.50, 3.51)* | 110.4 | 79.7 | 1.92 (1.21, 3.06)* |
|  | Quinolones | 31.2 | 22.1 | 2.10 (0.77, 5.78) | 32.7 | 29.7 | 1.58 (0.70, 3.54) |
| Antifungals, total of 3 subclasses | | 848.5 | 889.5 | 0.91 (0.74, 1.12) | 814.8 | 857.4 | 0.97 (0.89, 1.07) |
|  | Azoles | 559.8 | 661.3 | 0.68 (0.49, 0.95)* | 690.6 | 706.2 | 0.94 (0.84, 1.06) |
|  | Amphotericins | 158.0 | 120.1 | 1.65 (0.93, 2.92) | 94.7 | 63.5 | 1.83 (1.12, 2.97)* |
|  | Echinocandins | 130.7 | 108.1 | 1.41 (0.69, 2.87) | 29.5 | 87.7 | 0.43 (0.19, 0.96)* |
| Antivirals | | 70.7 | 145.6 | 0.59 (0.21, 1.62) | 114.8 | 151.2 | 0.67 (0.39, 1.13) |
| Vasopressors | | 27.6 | 5.2 | 8.07 (3.50, 18.6)* | 32.7 | 6.8 | 4.54 (2.08, 9.89)* |
|  | Dopamine | 19.9 | 2.9 | 9.03 (3.47, 23.5)* | 17.7 | 3.2 | 5.54 (2.05, 15.0)* |
|  | Other vasopressors | 8.4 | 3.6 | 5.76 (1.50, 22.0)* | 23.4 | 4.9 | 4.38 (1.72, 11.2)* |
| Blood Products, total | | 298.8 | 246.5 | 1.29 (1.09, 1.54)* | 238.7 | 221.5 | 1.06 (0.94, 1.19) |
|  | Platelets | 206.1 | 179.5 | 1.32 (1.05, 1.66)* | 150.2 | 144.5 | 1.03 (0.88, 1.21) |
|  | Packed RBC | 135.2 | 105.7 | 1.30 (1.06, 1.59)* | 144.9 | 106.8 | 1.20 (1.06, 1.36)* |
|  | Fresh frozen plasma | 6.8 | 4.9 | 1.41 (0.44, 4.56) | 12.4 | 3.3 | 3.03 (1.23, 7.48)* |
| Parenteral Nutrition | | 111.4 | 169.8 | 1.00 (0.50, 2.01) | 86.2 | 84.8 | 1.28 (0.76, 2.14) |
| Oxygen Therapy | | 40.9 | 13.3 | 5.14 (1.96, 13.5)* | 32.2 | 8.7 | 3.63 (1.79, 7.36)* |

All comparisons adjusted for patient age at diagnosis, race, sex, and insurance status at start of course; *statistically significant associations, p<0.05; Abbreviations: aIRR= adjusted rate ratio; CI=confidence interval; RBC=red blood cells; FFP= fresh frozen plasma

**eTable 2 (continued). Comparisons of resource utilization rates (per 1000 inpatient days) by discharge status for each treatment course**

|  |  | **Intensification I** | | | **Intensification II** | | |
| --- | --- | --- | --- | --- | --- | --- | --- |
|  |  | Discharge Status | |  | Discharge Status | |  |
|  |  | Early | Standard | aIRR (95% CI) | Early | Standard | aIRR (95% CI) |
| Antibiotics, total of 5 subclasses | | 1589.9 | 1030.4 | 1.52 (1.39, 1.67)* | 1862.1 | 1357.5 | 1.36 (1.25, 1.49)* |
|  | Beta lactam anti-*Pseudomonas* | 676.6 | 457.4 | 2.30 (1.96, 2.70)* | 714.8 | 582.0 | 1.57 (1.34, 1.84)* |
|  | Broad Gram-positive | 505.0 | 314.4 | 2.51 (2.09, 3.01)* | 585.0 | 416.9 | 1.79 (1.50, 2.15)* |
|  | Aminoglycosides | 211.0 | 139.3 | 2.29 (1.66, 3.15)* | 313.0 | 174.4 | 2.38 (1.68, 3.35)* |
|  | Carbapenems with anti-*Pseudomonas* activity | 161.5 | 85.8 | 2.86 (1.89, 4.33)* | 196.8 | 150.8 | 1.57 (1.02, 2.42)* |
|  | Quinolones | 35.7 | 33.5 | 1.65 (0.66, 4.12) | 52.5 | 33.4 | 1.59 (0.71, 3.58) |
| Antifungals, total of 3 subclasses | | 775.3 | 853.1 | 0.93 (0.84, 1.03) | 882.9 | 915.9 | 0.96 (0.87, 1.07) |
|  | Azoles | 662.4 | 702.1 | 0.97 (0.86, 1.09) | 649.7 | 692.8 | 0.91 (0.79, 1.06) |
|  | Amphotericins | 62.1 | 51.6 | 1.52 (0.91, 2.57) | 169.4 | 116.7 | 1.41 (0.94, 2.11) |
|  | Echinocandins | 50.8 | 99.3 | 0.38 (0.19, 0.74)* | 63.8 | 106.4 | 0.81 (0.39, 1.67) |
| Antivirals | | 116.2 | 170.1 | 0.70 (0.41, 1.19) | 109.0 | 165.4 | 0.62 (0.32, 1.21) |
| Vasopressors | | 40.6 | 9.2 | 5.67 (2.63, 12.2)* | 45.7 | 14.0 | 3.56 (1.65, 7.66)* |
|  | Dopamine | 28.9 | 5.7 | 6.55 (2.79, 15.4)* | 19.6 | 7.5 | 2.56 (1.08, 6.04)* |
|  | Other vasopressors | 26.6 | 5.4 | 6.04 (2.38, 15.4)* | 34.3 | 9.4 | 4.31 (1.82, 10.2)* |
| Blood Products, total | | 259.3 | 228.7 | 1.17 (0.99, 1.38) | 383.1 | 319.6 | 1.25 (1.12, 1.40)* |
|  | Platelets | 168.3 | 154.5 | 1.21 (0.96, 1.52) | 287.0 | 244.2 | 1.26 (1.09, 1.46)* |
|  | Packed RBC | 151.4 | 109.6 | 1.42 (1.18, 1.72)* | 171.5 | 133.8 | 1.32 (1.11, 1.56)* |
|  | Fresh frozen plasma | 5.6 | 2.2 | 4.31 (1.61, 11.5)* | 8.6 | 5.5 | 1.72 (0.56, 5.25)* |
| Parenteral Nutrition | | 73.7 | 99.4 | 1.22 (0.75, 1.98) | 109.0 | 88.3 | 1.23 (0.82, 1.85) |
| Oxygen Therapy | | 40.0 | 18.3 | 2.22 (1.10, 4.51)* | 46.3 | 16.2 | 2.85 (1.39, 5.83)* |

All comparisons adjusted for patient age at diagnosis, race, sex, and insurance status at start of course; *statistically significant associations, p<0.05; Abbreviations: aIRR= adjusted rate ratio; CI=confidence interval; RBC=red blood cells; FFP= fresh frozen plasma
